# Asymptotic dichotomy in a class of higher order nonlinear delay differential equations

**DOI:** 10.1186/s13660-018-1949-7

**Published:** 2019-01-07

**Authors:** Yunhua Ye, Haihua Liang

**Affiliations:** 1 School of Mathematics, Jiaying University, Meizhou, China; 2 School of Mathematics and Systems Science, Guangdong Polytechnic Normal University, Guangzhou, China

**Keywords:** 34K11, 34K25, Asymptotic behavior, delay differential equation, higher order differential equation, oscillation, Schwarz inequality

## Abstract

Employing a generalized Riccati transformation and integral averaging technique, we show that all solutions of the higher order nonlinear delay differential equation

$$ y^{(n+2)}(t)+p(t)y^{(n)}(t)+q(t)f\bigl(y\bigl(g(t)\bigr)\bigr)=0 $$

will converge to zero or oscillate, under some conditions listed in the theorems of the present paper. Several examples are also given to illustrate the applications of these results.

## Introduction

The objective of this paper is to investigate the oscillation and asymptotic behavior of solutions to the following higher order nonlinear delay differential equation:

1.1 $$ y^{ ( {n+2} )} ( t )+p(t)y^{ ( n )} ( t )+q(t)f ( {y \bigl(g(t)} \bigr)=0,\quad t\in I, $$

where *n* is a positive integer, $I= [ {a,+\infty } ) \subset \mathbb{R}$ ($a\ge 0$). The coefficients $p\in C^{1} ( {I,\mathbb{R}} )$ and $q\in C ( {I,\mathbb{R}} )$ satisfy $p(t)\ge 0$ and $q(t)>0$. The function $g\in C^{1}({I, \mathbb{R}})$ satisfies $0< g ( t )\le t$, ${g}' ( t ) \ge 0$, and $\mathop{\lim }_{t\to +\infty } g ( t )= + \infty $. The function $f\in C ( {\mathbb{R},\mathbb{R}} )$ satisfies $f ( u )/u\ge K$ ($u\ne 0$) for some positive constant *K*. Our attention is restricted to the solutions of equation (1.1) which exist on the interval *I* and satisfy $\sup_{t\ge T}|y(t)|>0$ for any $T \ge a$. We make a standing hypothesis that equation (1.1) possesses such solutions.

A solution of equation (1.1) is called oscillatory if it has arbitrarily large zeroes, otherwise it is called non-oscillatory. Equation (1.1) is called oscillatory if all its solutions are oscillatory. For solutions of some nonlinear delay differential equations, some interesting phenomena will occur. For example, some solutions are oscillatory, while there may exist some other solutions which are not oscillatory but will converge to zero as time approaches to infinity. We take a third-order delay differential equation as example. The equation

$$ y'''+2y'+y\biggl(t- \frac{\pi }{2}\biggr)=0 $$

has an oscillatory solution $y_{1}(t)=\sin (t)$ and a non-oscillatory solution $y_{2}(t)=e^{\lambda t}$, where $\lambda <0$ is a root to the characteristic function $\lambda ^{3}+2\lambda +e^{-\frac{\pi }{2} \lambda }=0$. Another example is that the delay differential equation $y'''+y(t-\tau )=0$ for $\tau >0$ is oscillatory if and only if $\tau e>3$. However, the corresponding equation $y'''+y=0$ has a non-oscillatory solution $y_{1}(t)=e^{-t}$ and two oscillatory solutions $y_{2}(t)=e^{\frac{t}{2}}\cos (\frac{\sqrt{3}t}{2})$ and $y_{3}(t)=e ^{\frac{t}{2}}\sin (\frac{\sqrt{3}t}{2})$. So for higher-order delay differential equation, people naturally ask the following question: under what conditions does the equation have oscillatory or asymptotic behavior? This is the topic we will study in this paper.

Since Sturm [20] introduced the concept of oscillation when he studied the problem of the heat transmission, the oscillation theory has been a very active area of research in the qualitative analysis of both ordinary and functional differential equations. Usually, a qualitative approach is concerned with the behavior of solutions of a given differential equation and does not seek explicit solutions. Since then, asymptotic and oscillatory properties of solutions to different equations, functional differential equations, and dynamical equation have attracted the attention of many researchers.

The oscillation and asymptotic behavior have extensive applications in the real world, the readers can refer to the monographs [1, 4, 6, 14], and [21] for more details. The problem of obtaining the oscillatory and asymptotic behavior of certain higher order nonlinear functional differential equations has been studied by a number of authors. The interested readers can see [3, 5, 9–11, 13, 17, 18], and the references cited therein.

There are many excellent works studying the oscillations and asymptotic behaviors of solutions to higher-order nonlinear delay differential equations, to list all of which is almost impossible. We just list some studies relating to our work below.

In 1971 and 1977, Ladas [12] and Magfoud [16] studied the oscillation of solutions to the equation

$$ x^{(n)}(t)+a(t)f\bigl(x\bigl(g(t)\bigr)\bigr)=0, $$

where $0< g(t)< t$, $g(t)\to \infty $ as $t \to +\infty $ and $a(t)>0$.

In 2008, the authors studied in [2] the following 2*n*-order nonlinear functional differential equation:

$$ \frac{d^{n}}{dt^{n}} \biggl(a(t) \biggl(\frac{d^{n}x(t)}{dt^{n}} \biggr)^{ \alpha } \biggr)+q(t)f \bigl(x\bigl(g(t)\bigr) \bigr)=0, $$

where *α* is the ratio of two positive odd integers. The oscillation theorems established in that paper extend a number of existing results.

In 2014, the oscillation and asymptotic behavior of solutions to the following nonlinear delay differential equation were studied in [15]:

$$ x^{(n+3)}(t)+p(t)x^{(n)}(t)+q(t)f\bigl(x\bigl(g(t)\bigr)\bigr) = 0. $$

In that paper, he used a generalized Riccati transformation and an integral averaging technique to study the sufficient conditions for the oscillations of differential equations. The goal of the present paper is to use a generalized method to study the oscillation and asymptotic behavior of solutions to the nonlinear delay differential equation (1.1). We need to carry out much more delicate analysis to overcome the difficulties in the proof. For the special case $n=1$, equation (1.1) reduces to the form

$$ {y}''' ( t )+p(t){y}' ( t )+q(t)f ( {y\bigl(g(t)} \bigr)=0, $$

which is exactly the main equation studied in [22] with $r_{1} (t )=1$ and $r_{2} ( t )=1$ as a special case. The third order differential equations arise in the study of entropy-flow phenomenon, problems of hydrodynamics, three-layer beams and so forth, see the monograph [7] and the references cited therein. It is interesting to study the oscillation and asymptotic behavior of general higher order differential equation (1.1), and we give generalizations of the former studies in [22].

The present paper is organized as follows. In Sect. 2, we present some lemmas which are useful in the proof of our main results. In Sect. 3, we carry out delicate analysis to give several oscillatory and asymptotic criteria for the higher order nonlinear delay differential equation (1.1). Noting that the delay $g(t)$ has the form $g(t)=t-\tau $ or the form $g(t)=at$ in many applications, therefore, in Sect. 4, we give two examples to illustrate the applications of our main theorems.

## Some preliminary lemmas

To give the main results of this paper, we first present and prove some useful lemmas. These lemmas play central roles in the proof of our new oscillation and asymptotic results in the next section.

### Lemma 2.1

*Suppose that the equation*

2.1 $$ {z}'' ( t )+p( t )z( t )=0 $$

*is non*-*oscillatory and suppose that* $y ( t )$ *is a non*-*oscillatory solution of equation* (1.1) *on* $[T,+\infty )$, *where* $T\ge a$. *Then there exists* $t_{0} \in [T,+\infty )$ *such that*, *for any* $t\ge t_{0} $, *we have* $y^{ ( n )}(t)>0$ *or* $y^{ ( n )}(t)<0$.

### Proof

We use the contradiction argument to prove this lemma. Suppose that $y ( t )$ is a non-oscillatory solution of equation (1.1). Without loss of generality, we may assume $y(t)>0$ and $y(g(t))>0$ for $t\ge t_{0} \ge T$. It is easy to see that $x(t)=-y^{ ( n )}(t)$ is the solution of the following second order delay differential equation:

2.2 $$ {x}'' ( t )+p ( t ) x ( t )=q(t)f \bigl( {y \bigl( {g(t)} \bigr)} \bigr) \quad \text{for } t \ge t_{0}. $$

Suppose that $z ( t )$ is a non-oscillatory solution of equation (2.1). Without loss of generality, we may assume $z(t)>0$ for $t\ge t_{0} $. Suppose that $x ( t )$ is an oscillatory solution to equation (2.2), *a* and *b* ($t_{0} < a< b$) are its two adjacent zero points of *x* such that ${x}'(a)\ge 0$ and ${x}'(b)\le 0$. From equation (2.1) and equation (2.2), we get

$$ \begin{aligned} z(t)q(t)f \bigl( {y \bigl( {g(t)} \bigr)} \bigr) &= z(t) \bigl\{ {{x}'' ( t )+p ( t ) x ( t )} \bigr\} \\ &= {x}'' ( t ) z(t)+p ( t ) x ( t ) z(t) = {x}'' ( t ) z(t)-{z}'' ( t ) x ( t ) \\ &= \bigl( {{x}'(t)z(t)-x(t){z}'(t)} \bigr)^{\prime }, \end{aligned} $$

i.e., we have

2.3 $$ \bigl( {{x}'(t)z(t)-x(t){z}'(t)} \bigr)^{\prime }=z(t)q(t)f \bigl( {y \bigl( {g(t)} \bigr)} \bigr). $$

Integrating from *a* to *b* on both sides of equation (2.3), we obtain

$$ 0\ge {x}'(b)z(b)-{x}'(a)z(a)= \int _{a}^{b} z(t)q(t)f\bigl(y\bigl(g(t)\bigr) \bigr)\,dt>0, $$

which leads to a contradiction, and we thus complete the proof of Lemma 2.1. □

### Lemma 2.2

*Suppose that equation* (2.1) *is non*-*oscillatory*, $y ( t )$ *is a non*-*oscillatory solution to equation* (1.1), *and there exists a constant* $T\ge a$ *such that* $y(t)y^{ ( n )}(t)>0$ *for* $t\ge T\ge a$. *Then* $y(t)y^{ ( {n+1} )} ( t )$ *is eventually positive*.

### Proof

Let $y ( t )$ be a non-oscillatory solution to equation (1.1) such that $y(t)y^{ ( n )}(t)>0$ for $t\ge T\ge a$. Without loss of generality, we may assume $y(t)>0$, $y(g(t))>0$, and $y^{ ( n )}(t)>0$ ($t\ge t_{0} \ge T$). Using equation (1.1), we know

2.4 $$ y^{ ( {n+2} )} ( t )< 0 \quad \text{for } t\ge T. $$

From the above inequality, we know $y^{ ( {n+1} )} ( t )$ is strictly monotonically decreasing in the interval $[T,+\infty )$, and therefore $y^{ ( {n+1} )} ( t )$ is eventually positive or eventually negative.

We use a contradiction argument to exclude the case that $y^{ ( {n+1} )} ( t )$ is eventually negative. In fact, if $y^{ ( {n+1} )} ( t )$ is eventually negative, without loss of generality, we may assume $y^{ ( {n+1} )} ( t )<0$ for $t\ge T$. By using the monotone decreasing of $y^{ ( {n+1} )} ( t )$, we have

2.5 $$ y^{ ( {n+1} )} ( t )\le y^{ ( {n+1} )} ( T ) \quad \text{for } t\ge T. $$

Integrating from *T* to *t* on both sides of inequality (2.5), we obtain

$$ y^{ ( n )} ( t )\le y^{ ( n )} ( T )+ ( {t-T} ) y^{ ( {n+1} )} ( T ) \quad \text{for } t\ge T. $$

By letting $t\to +\infty $ in the above inequality, we see $y^{ ( n )} ( t )\to -\infty $ and this leads to a contradiction. Therefore $y(t)y^{ ( {n+1} )} ( t )$ is eventually positive and hence we have completed the proof of Lemma 2.2.

By careful check of the proving process of Lemma 1.1 in [8], we obtain the following results which will be used in the following proof. □

### Lemma 2.3

*Assume that* $x\in C^{n} ( {[a,+\infty ),\mathbb{R}} )$ *such that* $x ( t )>0$, $x^{ ( n )} ( t ) \le 0$ *for* $t\ge a$, *and* $x^{ ( n )} ( t )$ *does not vanish identically on any interval* $[ {T,\infty } ) \subset [a,+\infty )$. *If* *n* *is even* (*or odd*), *then there exists* $l\in \{ {1, 3,\ldots , n-1} \}$ (*respectively* $l\in \{ {0,2,4,\ldots , n-1} \}$) *such that*, *for all sufficiently large* *t*, $x ( t ) x^{ ( j )} ( t )>0$ *for* $j=0, 1,\ldots , l$ *and* $( {-1} )^{n+j-1}x ( t ) x^{ ( j )} ( t )>0$ *for* $j=l+1, l+2,\ldots , n-1$. *Furthermore*, *if* $l\ge 1$, *then*

2.6 $$ \bigl\vert {{x}' \bigl( {g ( t )} \bigr)} \bigr\vert \ge \frac{g ^{l-1} ( t ) ( {t-g ( t )} ) ^{n-l-1}}{2^{l-1} ( {l-1} )! ( {n-l-1} )!} \bigl\vert {x^{ ( {n-1} )} ( t )} \bigr\vert $$

*for all sufficiently large* *t*.

### Lemma 2.4

*Suppose that the function* $x\in C^{2} ( {[T,+\infty ), \mathbb{R}} )$, *then both of the following statements hold*:

(i) *If* $x''(t) \le 0$ *and* ${x}' ( t )<0$ *for* $t\ge T$, *then* $\lim_{t\to +\infty } x(t)=-\infty $;
(ii) *If* $\lim_{t\to +\infty } x'(t)=\mu $, *where* $\mu >0$ *or* $\mu =+\infty $, *then* $\lim_{t\to +\infty } x(t) = +\infty $.

The statements are obvious, which can be easily checked, we therefore omit the proof here.

### Lemma 2.5

*Suppose that* *n* *is a positive integer*, $x\in C^{n}( {[T,+\infty ), \mathbb{R}})$, $g\in C^{1}({[T,+\infty ), \mathbb{R}})$, $x^{(n )}(t )<0$, $x(t )>0$, $g(t )< t$ *for* $t\ge T$ *and* $\lim_{t\to +\infty } x(t) \ne 0$, $\lim_{t\to +\infty } g(t )=+\infty $. *Then both of the following statements hold*:

(i) *If* $n=1$, *then there exist* $\lambda >0$ *and* $T'>0$ *such that* $x({g(t)})>x(t )>\lambda $ *for* $t\ge T'$;
(ii) *Suppose that* $n\ge 2$, *then there exist* $\lambda >0$ *and* ${T}'>0$ *such that* $0< x^{({n-1})}(t )<\lambda <x(t )$, $x({g(t )})> \lambda $ *for* $t\ge {T}'$.

### Proof

Suppose first $n=1$. Then $x'(t)<0$ and $x(t)>0$ for $t\ge T$. Hence the function $x(t )>0$ is monotonically decreasing on the interval $[T,+\infty )$. Using the monotone bounded theorem, we know the limit $\lim_{t\to +\infty } x(t)$ exists and we denote $\lim_{t\to +\infty } x(t)=\lambda $. Using the monotonicity of $x(t)$ again, we know $x(t)>\lambda $ for $t\ge T$. Noting $g(t )< t$ and $\lim_{t\to +\infty } g(t)=+\infty $, we can find ${T}'\ge T$ such that $t>g(t)\ge T$ for $t\ge T'$. Since the function $x(t)$ is monotonically decreasing, we deduce that $x(g(t))>x(t)>\lambda $ for $t\ge T'$ and we have proved statement (i).

Now we suppose that $n\ge 2$. Since $x^{(n)}(t)<0$ for $t\ge T$, we deduce that $x^{({n-1})}(t)$ is monotonically decreasing on the interval $[T,+\infty )$, and therefore $x^{({n-1})}(t)$ is eventually positive or eventually negative. We use a contradiction argument to exclude the case for eventual negativeness. In fact, if $x^{({n-1} )}(t)$ is eventually negative, by using the fact that $x^{(n )}(t)<0$ for $t\ge T$ and Lemma 2.4, we obtain $\lim_{t\to +\infty } x ^{({n-2})}(t)=-\infty $. Similarly, by using the induction method, we know $x(t)\to -\infty $ as $t\to +\infty $, which is a contradiction with $x(t)>0$. Hence $x^{({n-1} )}(t)$ is eventually positive. Without loss of generality, we may assume $x^{({n-1} )}(t)>0$ for $t\ge T$ and $x^{({n-1} )}(t)$ is monotonically decreasing on the interval $[T,+\infty )$. By using the monotone bounded theorem, we know

$$ \lim_{t\to +\infty } x^{({n-1} )}(t)=\mu \ge 0. $$

From Lemma 2.3 we know that $x(t)$ is eventually strictly monotonous and $x(t)>0$ for $t\ge T$. Since $\lim_{t\to +\infty } x(t) \ne 0$, we assume $\lim_{t\to +\infty } x(t)=\lambda _{0} $, where $\lambda _{0} >0$ or $\lambda _{0} =+\infty $. We divide the proof of statement (ii) into two cases. We first deal with the case for $\mu =0$. Let $\lambda \in ({0, \lambda _{0} })$, then there exists $T_{1} \ge T$ such that $0< x^{({n-1})}(t)<\lambda <x(t)$ for $t\ge T_{1} $. Since $\lim_{t\to +\infty } g(t )=+\infty $, then there exists ${T}'\ge T_{1} $ such that $g(t )\ge T$ for $t\ge {T}'$. Therefore we know $x({g(t)})>\lambda $ and $0< x^{({n-1})}(t)<\lambda <x(t)$ for $t\ge {T}'$, and hence we have proved that statement (ii) holds in this case. For the second case $\lim_{t\to +\infty } x^{( {n-1} )}(t)=\mu >0$, we repeatedly use Lemma 2.4 to deduce $\lambda _{0} =+\infty $. If we let $\lambda \in ({\mu ,+\infty })$, then there exists ${T}'\ge T$ such that $0< x^{({n-1})}(t )<\lambda <x(t)$ and $x({g(t)})>\lambda $ for $t\ge {T}'$. Statement (ii) also holds in this case, and we have completed the proof of Lemma 2.5. □

## Asymptotic dichotomy

In this section we present some sufficient conditions which guarantee that every solution to equation (1.1) oscillates or converges to zero as *t* approaches to infinity. Throughout this section we will suppose that one of the following conditions holds:

3.1 $$ \textstyle\begin{cases} \limsup_{t\to + \infty } \int _{T}^{t}\int _{v}^{t}\int _{s} ^{+\infty } (\mathit{Kq}(u)-p'(s) )\,du\,ds\,dv = +\infty , \\ \limsup_{t\to + \infty } \int _{T}^{t}\int _{v}^{t} p(s)\,ds\,dv < +\infty , \end{cases} $$

or

3.2 $$ \limsup_{t\to + \infty } \int _{T}^{t} \bigl(\mathit{Kq}(s)-p'(s) \bigr)\,ds=+\infty , $$

where the integrand $\mathit{Kq}(t)-p'(t)\ge 0$ for $t\in [a,+\infty )$ and does not vanish identically on any subinterval of $[a,+\infty )$.

### Theorem 3.1

*Suppose that equation* (2.1) *is non*-*oscillatory and condition* (3.1) *or* (3.2) *holds*. *We further assume that there exists a positive differentiable function* *ρ* *such that*

3.3 $$ \begin{aligned}[b] &\limsup_{t\to + \infty } \int _{T}^{t} \biggl\{ K\rho (s)q(s)- \frac{2^{n}n!}{4g ^{n}(s)g'(s)\rho (s)} \\ &\quad {}\times \bigl(\rho '(s)-p(s)\rho (s) \bigl(s-g(s)\bigr) \bigr)^{2} \biggr\} \,ds=+ \infty . \end{aligned} $$

*In addition*, *for* $n\ge 2$, *we further assume* ${p}'(t)\ge 0$ *for* $t\in [a,+\infty )$. *Then any solution* $y(t)$ *of equation* (1.1) *is oscillatory or converges to zero*, *i*.*e*., $y(t)\to 0$ *as* $t\to +\infty $.

### Proof

Suppose that $y(t)$ is a non-oscillatory solution of equation (1.1) on the interval $[T,+\infty )$, where $T\ge a$. Without loss of generality, we may assume $y(t)>0$ and $y(g(t))>0$ for $t\ge t_{0} \ge T$. By Lemma 2.1, there exists $t_{1} $ such that $y^{(n)}(t)>0$ or $y^{(n)}(t)<0$ for $t\ge t_{1} \ge t_{0} $.

If $y^{(n)}(t)>0$ for $t\ge t_{1} $, by using Lemma 2.2, we know that there exists $t_{2} \ge t_{1} $ such that $y^{({n+1})}(t)>0$ for $t\ge t_{2} $. We now define a function

3.4 $$ \omega ( t )=\rho ( t ) \frac{y^{ ({n+1} )} ( t )}{y ( {g ( t )} )}. $$

Since $\rho (t)$ is positive, we know that the function $\omega (t)>0$. Using equation (1.1), we know $y^{(n+2)} ( t )<0$ for $t\ge t_{2} $. Since $y(t)>0$, $y^{(n)}(t)>0$, $y^{ ( {n+1} )} ( t )>0$, and $y^{ ( n )} ( t )>0$ for $t\ge t_{2} $, then we can replace *n* with $n+2$ and use Lemma 2.3 to conclude $l=n+1$. Since $\lim_{t \to +\infty } g(t)= + \infty $ by our assumption, we may find $t_{3} \ge t_{2} $ such that, for $t\ge t_{3} $, we have $g ( t )\ge t_{2} $. Hence, for $t\ge t_{3} $, we obtain

3.5 $$ y^{ ( n )} ( t )\ge y^{ ( n )} ( t )-y^{ ( n )} \bigl( {g ( t )} \bigr)\ge y^{ ( {n+1} )} ( t ) \bigl( {t-g ( t )} \bigr) . $$

By using equation (1.1), (3.4), (3.5), and Lemma 2.3, we know that, for $t\ge t_{3} $,

$$ \begin{aligned}{\omega }'(t)={}&\omega (t) \frac{{\rho }'(t)}{\rho (t)}-\frac{\rho (t)}{y( {g(t)})} \bigl\{ p(t)y^{(n)}(t)+q(t)f ( y \bigl(g(t) \bigr) \bigr\} - \omega (t)\frac{{y}'( {g ( t )}){g}'(t)}{y({g(t)})} \\ \le{} & {-}K\rho ( t ) q(t)+\omega ( t ) \frac{ {\rho }' ( t )}{\rho ( t )}-\frac{\rho ( t ) p(t)y^{ ( n )} ( t )}{y ( {g ( t )} )}- \omega ( t ) \frac{g ^{n} ( t ) }{2^{n}n!}\frac{y^{ ( {n+1} )} ( t ) {g}' ( t )}{y ( {g ( t )} )} \\ \le {}& {-}K\rho ( t ) q(t)+\omega ( t ) \frac{ {\rho }' ( t )}{\rho ( t )}-\frac{\rho ( t ) p(t)y^{ ( n )} ( t )}{y ( {g ( t )} )}- \omega ^{2} ( t ) \frac{g ^{n} ( t ) {g}' ( t )}{2^{n}n!\rho ( t )} \\ \le {}& {-}K\rho (t)q(t)+\omega (t)\frac{{\rho }'(t)}{\rho (t)}-\frac{ \rho (t)p(t)y^{({n+1}}(t)({t-g(t)})}{y({g(t)})}-\omega ^{2}(t)\frac{g ^{n}(t){g}'(t)}{2^{n}n!\rho (t)} \\ \le {}&{-}K\rho (t)q(t)+\omega (t) \biggl\{ {\frac{{\rho }'(t)}{\rho (t)}-p(t) \bigl( {t-g(t)} \bigr)} \biggr\} -\omega ^{2}(t) \frac{g^{n}(t){g}'(t)}{2^{n}n!\rho (t)} \\ = {}& {-}K\rho (t)q(t)-\frac{g^{n}(t){g}'(t)}{2^{n}n!\rho (t)} \biggl\{ {\omega ^{2}(t)- \frac{2^{n}n!\rho (t)}{g^{n}(t){g}'(t)}} \\ & {} \times \biggl( {\frac{{\rho }' ( t )}{\rho ( t )}-p(t) \bigl( {t-g ( t )} \bigr)} \biggr) \omega ( t ) \biggr\} \\ ={} &{-}K\rho (t)q(t)-\frac{g^{n}(t){g}'(t)}{2^{n}n!\rho (t)} \biggl[{\omega (t) -\frac{2^{n}n!}{2g ^{n}(t){g}'(t)}} { \times \bigl({{\rho }'(t)-p(t)\rho (t) \bigl({t-g(t)}\bigr)} \bigr)} \biggr]^{2} \\ &{}+\frac{2^{n}n!}{4g^{n} ( t ) {g}' ( t ) \rho ( t )} \bigl( {{\rho }' ( t )-p(t)\rho ( t ) \bigl( {t-g ( t )} \bigr)} \bigr)^{2} \\ \le{} & {-} \biggl\{ {K\rho ( t ) q(t)-\frac{2^{n}n!}{4g ^{n} ( t ) {g}' ( t ) \rho ( t )}} { \bigl( {{\rho }' ( t )-p(t) \rho ( t ) \bigl( {t-g ( t )} \bigr)} \bigr)^{2}} \biggr\} . \end{aligned} $$

Integrating from $t_{3} $ to *t* on both sides of the above inequality, we deduce that, for $t\ge t_{3} $,

$$ \begin{aligned} & \int _{t_{3}}^{t} \biggl\{ K\rho (s)q(s)- \frac{2^{n}n!}{4g^{n}(s)g'(s) \rho (s)} \bigl(\rho '(s)-p(s)\rho (s) \bigl(s-g(s)\bigr) \bigr)^{2} \biggr\} \,ds \\ &\quad \le \omega ({t_{3} } )-\omega ( t )\le \omega ({t_{3}} ), \end{aligned} $$

which is a contradiction with (3.3) valuing at $T=t_{3} $.

Therefore we know $y^{(n)}(t)<0$ for $t\ge t_{1} $. Noting that $y(t)>0$ and $y(g(t))>0$, we consider $y^{(n+1)}(t)$ is either eventually negative or eventually positive.

First we prove Theorem 3.1 under condition (3.1). We now exclude the case that $y^{(n+1)}(t)$ is eventually negative. In fact, if $y^{(n)}(t)<0$ for $t\ge t_{1} $. By using Lemma 2.4, we obtain $\lim_{t\to +\infty } y^{ ( {n-1} )}(t)=- \infty $. Similarly, by induction on *n*, we deduce that $y(t)\to - \infty $ as $t\to +\infty $, which is a contradiction with $y(t)>0$. Hence the function $y^{ ( {n+1} )}(t)$ is eventually positive, and hence there exists $t_{4} \ge t_{1} $ such that $y^{ ( {n+1} )}(t)>0$ for $t\ge t_{4} $.

We prove $y(t)\to 0$ as $t\to +\infty $ by using a contradiction argument. Otherwise, we assume $\lim_{t\to +\infty } y(t)\ne 0$. We divide the proof into two cases with respect to *n*.

First we prove for the special case $n=1$. Then we have ${y}''(t)>0$, ${y}'(t)<0$, $y(t)>0$ for $t\ge t_{4} $. By Lemma 2.5, we know there exist $\mu >0$ and $t_{5} \ge t_{4} $ such that $y ( {g ( t )} )>y ( t )>\mu $ for $t\ge t _{5} $. By equation (1.1), we get

3.6 $$ {y}''' ( t )+ \bigl( {p(t)y ( t )} \bigr) ^{\prime }+q(t)f ( {y\bigl(g(t)} \bigr)-{p}'(t)y ( t )=0. $$

Integrating from *s* to *t* on both sides of (3.6), we get

$$ \begin{aligned} {y}''(s)+p(s)y(s)&= {y}''(t)+p(t)y(t)+ \int _{s}^{t} y(u) \biggl(q(u) \frac{f(y(g(u)))}{y(u)}-p'(u) \biggr)\,du \\ &\ge \mu \int _{s}^{t} \biggl(q(u)\frac{f(y(g(u)))}{y(g(u))}-p'(u) \biggr)\,du \\ &\ge \mu \int _{s}^{t} \bigl(\mathit{Kq}(u)-p'(u) \bigr)\,du \end{aligned} $$

for $t\ge s\ge t_{5} $. Letting $t\to +\infty $ in the above inequality, we obtain

3.7 $$ {y}'' ( s )+p(s)y(s)\ge \mu \int _{s}^{+\infty } \bigl(\mathit{Kq}(u)-p'(u) \bigr) \,du. $$

We integrate from *v* to *t* on both sides of inequality (3.7) to get

$$ \begin{aligned} -{y}'(v)+y(v) \int _{v}^{t} p(s)\,ds &\ge y'(t)-y'(v)+ \int _{v}^{t}p(s)y(s)\,ds \\ &\ge \mu \int _{v}^{t} \int _{s}^{+\infty } \bigl(K q(u)-{p}'(u) \bigr) \,du\,ds. \end{aligned} $$

Integrating from $t_{5} $ to *t* on both sides of the above inequality, we obtain

3.8 $$ y ( t )\le y ( t_{5} )+y (t_{3} ) \int _{t_{5}} ^{t} \int _{v}^{t} p(s)\,ds\,dv-\mu \int _{t_{3}} ^{t} \int _{v} ^{t} \int _{s}^{+\infty } \bigl(K q(u)-{p}'(u) \bigr) \,du\,ds\,dv. $$

Combining (3.1) and (3.8), we know $y ( t )<0$ for sufficiently large *t*, which leads to a contradiction with $y(t)>0$.

We now prove for the case $n\ge 2$. Then we know $y^{ ( n )}(t)<0$, $y(t)>0$ for $t\ge t_{1} $ and $\lim_{t\to +\infty } y(t)\ne 0$. By Lemma 2.5, we know there exist $\lambda _{1} >0$ and $t_{6} \ge t_{1} $ such that $0< y^{ ( {n-1} )} ( t )<\lambda _{1} <y ( t )$, $y ( {g ( t )} )>\lambda _{1} $ for $t\ge t_{6} $.

By equation (1.1), we deduce that, for $t\ge s\ge t_{6} $,

$$ \begin{aligned} y^{ ( {n+1} )} ( s )+p(s)y^{ ( {n-1} )} ( s ) ={}& y^{ ( {n+1} )} ( t )+p(t)y ^{ ( {n-1} )} ( t ) \\ &{}+ \int _{s}^{t} \biggl( y\bigl(g(u)\bigr)q(u) \frac{f(y(g(u)))}{y(g(u))}-p'(u)y ^{(n-1)}(u) \biggr)\,du \\ \ge{} & \lambda _{1} \int _{s}^{t} \bigl( K q(u)-p'(u) \bigr) \,du, \end{aligned} $$

i.e., for $t\ge v\ge t_{6} $, we know

3.9 $$ y^{(n+1)}(s)+p(s)y^{(n-1)}(s)\ge \lambda _{1} \int _{s}^{+\infty } \bigl( K q(u)-p'(u) \bigr) \,du. $$

Integrating from *v* to *t* on both sides of (3.9), we obtain

$$ \begin{aligned} -y^{(n)} ( v )+y^{(n-1)} ( v ) \int _{v}^{t} p(s)\,ds &\ge y^{(n)}(t)-y^{(n)}(v)+ \int _{v}^{t} p(s)y^{(n-1)}(s)\,ds \\ &\ge \lambda _{1} \int _{v}^{t} \int _{s}^{+\infty } \bigl( \mathit{Kq}(u)-p'(u) \bigr) \,du\,ds. \end{aligned} $$

We integrate from $t_{6} $ to *t* on both sides of the above inequality to get

3.10 $$ \begin{aligned}[b] y^{(n-1)}(t)\le {}& y^{(n-1)} ( t_{6} )+y^{ ( n-1 )} (t_{6} ) \int _{t_{6}}^{t} \int _{v}^{t} p(s)\,ds\,dv \\ &{} -\lambda _{1} \int _{t_{6}} ^{t} \int _{v}^{t} \int _{s}^{+\infty } \bigl( \mathit{Kq}(u)-p'(u) \bigr) \,du\,ds\,dv. \end{aligned} $$

Combining (3.1) and (3.10), we deduce that $y^{(n-1)}(t)<0$ for sufficiently large *t*, which also leads to a contradiction.

If $y^{ ( {n+1} )}(t)$ does not change sign, then $y^{ ( {n+1} )}(t)$ will not be eventually positive or eventually negative, which contradicts with $y^{ ( n )}(t)<0$.

In the following, we prove Theorem 3.1 under condition (3.2). Then we know $y^{ ( n )}(t)<0$ for $t\ge t_{1} $. First we consider the case $n=1$. By Lemma 2.5, we know $\mu _{2} >0$ and there exists $t_{7} \ge t_{1} $ such that $y ( {g ( t )} )>y ( t )> \mu _{2} $ for $t>t_{7}$.

We integrate from $t_{7} $ to *t* on both sides of equation (1.1) to get

$$ \begin{aligned}[b] 0= {}& {y}'' ( t )-{y}'' ( {t_{7} } )+p ( t )y ( t )-p ( {t_{7} } )y ( {t_{7} } ) \\ &{} - \int _{t_{7} }^{t} {{p}'(u)y ( u )\,du} + \int _{t_{7}} ^{t} {q(u)f ( {y\bigl(g(u)} \bigr)\,du}. \end{aligned} $$

Therefore we have

$$ \begin{aligned} {-}{y}'' ( t )+{y}'' ( {t_{7} } )-p ( t )y ( t )+p ( {t_{7} } )y ( {t_{7} } ) & = \int _{t_{7} }^{t} {y ( u ) \biggl( {q(u) \frac{f ( {y(g(u)} )}{y ( u )}-{p}'(u)} \biggr)\,du} \\ & \ge \mu _{2} \int _{t_{7} }^{t} { \biggl( {q(u)\frac{f ( {y(g(u)} )}{y(g(u)}-{p}'(u)} \biggr)\,du} \\ & \ge \mu _{2} \int _{t_{7}}^{t} { \bigl( {K q(u)-{p}'(u)} \bigr)\,du}. \end{aligned} $$

From the above inequality, we easily obtain

3.11 $$ {y}'' ( t )\le H_{1} -\mu _{2} \int _{t_{7} }^{t} { \bigl( {K q(u)-{p}'(u)} \bigr)\,du} -p ( t )y ( t ), $$

where $H_{1}$ is a constant. Combining (3.2) and (3.11), we conclude that ${y}'' ( t )$ is eventually negative.

Consider the case that $n\ge 2$. By Lemma 2.5, there exist $\lambda _{2} >0$ and $t_{8} \ge t_{1} $ such that $0< y^{ ( {n-1} )} ( t )<\lambda _{2} <y ( t )$ and $y ( {g ( t )} )>\lambda _{2} $ for $t\ge t _{8} $.

We integrate from $t_{8} $ to *t* on both sides of equation (1.1) to get

3.12 $$\begin{aligned} & y^{ ( {n+1} )} ( {t_{8} } )+p ( {t_{8} } )y^{ ( {n-1} )} ( {t_{8} } ) \\ &\quad = y^{ ( {n+1} )} ( t )+p(t)y^{ ( {n-1} )} ( t )+ \int _{t_{8} }^{t} { ( {y\biggl(g(u)q(u) \frac{f ( {y(g(u)} )}{y(g(u)}-{p}'(u)y^{ ( {n-1} )} ( u )} \biggr)\,du} \\ &\quad \ge y^{ ( {n+1} )} ( t )+ \int _{t_{8} }^{t} { ( {y\biggl(g(u)q(u) \frac{f ( {y(g(u)} )}{y(g(u)}-{p}'(u)y ^{ ( {n-1} )} ( u )} \biggr)\,du} \\ &\quad \ge y^{ ( {n+1} )} ( t )+\lambda _{2} \int _{t_{8} }^{t} { \bigl( {K q(u)-{p}'(u)} \bigr)\,du}, \end{aligned}$$

i.e.,

3.13 $$ y^{ ( {n+1} )} ( t )\le H_{2} -\lambda _{2} \int _{t_{8} }^{t} { \bigl( K {q(u)-{p}'(u)} \bigr)\,du}, $$

where $H_{2} $ is another constant. Combining (3.2) and (3.13), we know $y^{ ( {n+1} )} ( t )$ is eventually negative. Noting that $y^{ ( n )}(t)<0$ for $t\ge t_{1} $, we use Lemma 2.4 to conclude that $\lim_{t\to +\infty } y^{ ( {n-1} )} ( t )=- \infty $. Similarly, we can use the induction method to obtain $\lim_{t\to +\infty } y ( t )=-\infty $. This is a contradiction and we finish the proof of Theorem 3.1.

In the following, we prove a new asymptotic dichotomy for equation (1.1) under the so-called Philos-type integral averaging conditions. Following the literature [19], we first introduce a class of functions ℜ. We define two sets $D_{0} =\{(t,s): t>s \ge T \}$ and $D=\{(t,s):t\ge s \ge T\}$.

If a function $H\in C(D,\mathbb{R})$ satisfies

(i) $H(t,t)=0$ for $t\ge T$ and $H(t,s)>0$ for $(t,s)\in D_{0} $;
(ii) *H* has a continuous and non-positive partial derivative on $D_{0}$ with respect to the second variable such that $- \frac{{\partial H ( {t, s} )} }{{\partial s}}=h(t,s) \sqrt[2]{H(t,s)}$ for $(t,s)\in D_{0}$;

then *H* is said to belong to ℜ. □

### Theorem 3.2

*Suppose that equation* (2.1) *is non*-*oscillatory and condition* (3.1) *or* (3.2) *holds*. *We further suppose that there exist two functions* $H\in \Re $ *and* $0<\rho \in C^{1} ( { [ {T,\infty } )} )$ *satisfying*

3.14 $$ \limsup_{t\to + \infty } \frac{1}{H ( t, T )} \int _{T}^{t} \biggl( \mathit{KH}(t,s)\rho (s)q(s)- \frac{Q^{2}(t,s)}{4W(s)H(t,s)} \biggr)\,ds = +\infty , $$

*where*

3.15 $$\begin{aligned}& Q ( {t, s} )=h ( {t, s} ) -\sqrt[2]{H ( {t, s} )} \gamma ( s ), \end{aligned}$$

3.16 $$\begin{aligned}& \gamma ( t )=\frac{{\rho }' ( t )}{\rho ( t )}-p(t) \bigl( {t-g ( t )} \bigr), \end{aligned}$$

3.17 $$\begin{aligned}& W ( t )=\frac{g^{n} ( t ) {g}' ( t )}{2^{n}n! \rho ( t )}. \end{aligned}$$

*For the case* $n\ge 2$, *we further assume* ${p}' ( t ) \ge 0$ *for* $t\in [ {a,+\infty } )$. *Then any solution* $y ( t )$ *of equation* (1.1) *is oscillatory or* $y(t)\to 0$ *as* $t\to +\infty $.

### Proof

Suppose that $y ( t )$ is a non-oscillatory solution of equation (1.1) on the interval $[T,+\infty )$, where $T\ge a$. Without loss of generality we may assume $y(t)>0$ and $y(g(t))>0$ for $t\ge t_{0} \ge T$. By Lemma 2.1, we deduce that $y^{ ( n )}(t)>0$ or $y^{ ( n )}(t)<0$ for $t\ge t_{1} \ge t_{0} $.

If $y^{ ( n )}(t)>0$ for $t\ge t_{1} $, then by Lemma 2.2, we know $y^{ ( {n+1} )} ( t )>0$.

We define $\omega ( t )$ as in (3.4), i.e.,

$$ \omega (t)=\rho (t)\frac{y^{({n+1})}(t)}{y({g(t)})} $$

for $t\ge t_{1} $. It is easy to see that $\omega ( t )>0$. Using equation (1.1), we get $y^{ ( {n+2} )} ( t )<0$ for $t\ge t_{1} $. Note that $y(t)>0$, $y^{ ( n )}(t)>0$, $y^{ ( {n+1} )} ( t )>0$, and $y^{ ( {n+2} )} ( t )<0$ for $t\ge t_{1} $. We replace *n* with $n+2$ and use Lemma 2.3 to conclude that $l=n+1$. Since $\lim_{t\to +\infty }g(t)=+\infty $, then we may find $t_{2} \ge t_{1} $ such that $g(t)\ge t_{1} $ for $t\ge t_{2} $. Therefore a relation similar to (3.5) holds for $t\ge t_{2} $, i.e.,

$$ y^{(n)}(t)\ge y^{(n)}(t)-y^{(n)}\bigl(g(t)\bigr)\ge y^{(n+1)}(t) \bigl(t-g(t) \bigr). $$

Combining (1.1), (3.4), the above inequality, and Lemma 2.3, it follows that

3.18 $$ \begin{aligned}[b] {\omega }'(t) &= \omega (t)\frac{{\rho }'(t)}{\rho (t)}-\frac{\rho (t)}{y(g(t))} \bigl\{ p(t)y^{(n)}(t) +q(t)f\bigl(y\bigl(g(t)\bigr)\bigr) \bigr\} -\omega (t)\frac{{y}'( {g ( t )}){g}'(t)}{y({g(t)})} \\ &\le -K\rho (t)q(t)+\omega (t)\frac{{\rho }'(t)}{\rho (t)}-\frac{ \rho (t)p(t)y^{(n)}(t)}{y({g(t)})}-\omega (t) \frac{g^{n}(t)y^{({n+1})}(t) {g}'(t)}{2^{n}y({g(t)})n!} \\ &\le -K\rho ( t ) q(t)+\omega ( t ) \frac{ {\rho }' ( t )}{\rho ( t )}-\frac{\rho ( t ) p(t)y^{ ( n )} ( t )}{y ( {g ( t )} )}- \omega ^{2} ( t ) \frac{g ^{n} ( t ) {g}' ( t )}{2^{n}n!\rho ( t )} \\ &\le -K\rho (t)q(t)+\omega (t)\frac{{\rho }'(t)}{\rho (t)}-\frac{ \rho (t)p(t)y^{({n+1})}(t)({t-g(t)})}{y({g(t)})} -\omega ^{2}(t)\frac{g ^{n}(t){g}'(t)}{2^{n}n!\rho (t)} \\ &\le -K\rho ( t ) q(t)+\omega ( t ) \biggl( {\frac{{\rho }' ( t )}{\rho ( t )}-p(t) \bigl( {t-g ( t )} \bigr)} \biggr)-\omega ^{2} ( t ) \frac{g^{n} ( t ) {g}' ( t )}{2^{n}n! \rho ( t )} \\ &=-K\rho (t) q(t)- \{ {\omega ^{2} ( t ) \biggl\{ {\biggl({ \frac{g^{n} ( t ) {g}' ( t )}{2^{n}n! \rho ( t )}}\biggr)} } {-\omega (t) \biggl( { \frac{{\rho }'(t)}{\rho (t)}-p(t) \bigl( {t-g(t)} \bigr)}\biggr)} \biggr\} . \end{aligned} $$

Since

$$ \gamma ( t )=\frac{{\rho }' ( t )}{\rho ( t )}-p(t) \bigl( {t-g ( t )} \bigr) $$

and

$$ W ( t )=\frac{g^{n} ( t ) {g}' ( t )}{2^{n}n! \rho ( t )}, $$

we carry out the estimates of inequality (3.18) and obtain

$$\begin{aligned} & \int _{t_{2}} ^{t} \mathit{KH}(t,s)\rho (s)q(s)\,ds \\ &\quad \le \int _{t_{2}}^{t} H(t,s) \bigl[-{\omega }'(s)+\gamma (s)\omega (s)-W(s) \omega ^{2}(s) \bigr]\,ds \\ &\quad =-H(t,s)\omega (s)\vert _{t_{2}} ^{t} + \int _{t_{2}}^{t} \biggl\{ \frac{ \partial H(t,s)}{\partial s}\omega (s)+H(t,s) \bigl[\gamma (s)\omega (s)-W(s) \omega ^{2}(s) \bigr] \biggr\} \,ds \\ &\quad = H(t,t_{2})\omega (t_{2})- \int _{t_{2}}^{t} \bigl\{ \omega ^{2}(s)W(s)H(t,s) \\ &\qquad {} + \omega (s) \bigl(h(t,s)\sqrt[2]{H(t,s)} -H(t,s)\gamma (s) \bigr) \bigr\} \,ds \\ &\quad = H(t,t_{2})\omega (t_{2})- \int _{t_{2}}^{t} W(s)H(t,s) \biggl\{ \omega ^{2}(s) \\ &\qquad {} +\omega (s)\frac{ (h(t,s)\sqrt[2]{H(t,s)}-H(t,s)\gamma (s) )}{W(s)H(t,s)} \biggr\} \,ds \\ & \quad =H(t,t_{2})\omega (t_{2})- \int _{t_{2}}^{t} W(s)H(t,s) \biggl(\omega (s)+ \int _{t_{2}}^{t} \frac{ ( {h(t,s)-\sqrt[2]{H(t,s)}\gamma (s)} )^{2}}{4W(s)}\,ds \\ &\qquad {}+\frac{ (h(t,s)\sqrt[2]{H(t,s)}-H(t,s)\gamma (s))}{2W(s)H(t,s)} \biggr)^{2} \,ds . \end{aligned}$$

Therefore

3.19 $$ \int _{t_{2}}^{t} \mathit{KH}(t,s)\rho (s ) q (s ) \,ds \le H (t, t_{2} ) \omega (t_{2} )+ \int _{t_{2}}^{t}\frac{Q^{2}(t,s)}{4W(s)}\,ds, $$

where $Q(t,s)=h(t,s) -\sqrt[2]{H ( {t, s} )} \gamma (s )$.

From inequality (3.19), we deduce that

3.20 $$ \frac{1}{H(t,t_{2})} \int _{t_{2}} ^{t} \biggl(\mathit{KH}(t,s)\rho (s)q(s)- \frac{Q ^{2}(t,s)}{4W (s ) } \biggr)\,ds \le \omega (t_{2}), $$

which contradicts with (3.14). The remaining proof is similar to the proof of Theorem 3.1, and we omit the details here. Therefore we have completed the proof of Theorem 3.2. □

### Theorem 3.3

*Suppose that equation* (2.1) *is non*-*oscillatory and condition* (3.1) *or* (3.2) *holds*. *Assume further that there exist two functions* $H\in \Re $ *and* $0< \rho \in C^{1}({[ {T,\infty })})$ *such that*

3.21 $$ 0< \inf_{s\ge T} \biggl[\lim_{t\to +\infty } \inf \frac{H ({t, s} )}{H ( {t, T} )} \biggr]\le +\infty $$

*and*

3.22 $$ \limsup_{t\to + \infty } \frac{1}{H ( t, T )} \int_{T}^{t} \frac{Q^{2}(t,s)}{W(s)}\,ds< +\infty $$

*hold for all* *T*, *where* $W ( t )$ *is defined as* (3.17) *in Theorem* 3.2. *In addition*, *there exists* $\varPsi \in C ({ [ {a,\infty } ),\mathbb{R}} )$ *such that*

3.23 $$ \limsup_{t\to + \infty } \int _{T}^{t} \varPsi ^{2}_{+}(s) W ( s ) \,ds=+\infty $$

*and*

3.24 $$ \limsup_{t\to + \infty } \frac{1}{H ( t, T )} \int _{T}^{t} \biggl[ K\rho (s)H(t,s) q ( s )- \frac{Q^{2} (t, s )}{4W ( s )} \biggr]\,ds \ge \sup_{t\ge T} \varPsi (t), $$

*where* $\varPsi _{+} ( t )=\max \{ {\varPsi (t), 0} \}$.

*Then any solution* $y(t)$ *of equation* (1.1) *is oscillatory or satisfies* $y ( t )\to 0$ *as* $t\to +\infty $.

### Proof

Suppose that $y ( t )$ is a non-oscillatory solution of equation (1.1) on the interval $[T,+\infty )$, where $T\ge a$. Without loss of generality, we may assume that $y(t)>0$ and $y(g(t))>0$ hold for $t\ge t_{0} \ge T$. By Lemma 2.1, we deduce that $y^{ ( n )}(t)>0$ or $y^{ ( n )}(t)<0$ for $t\ge t_{1} \ge t_{0} $.

If $y^{(n)}(t)>0$ for $t\ge t_{1} $. From Lemma 2.2, we know $y^{ ( {n+1} )} ( t )>0$. We define $\omega ( t )$, $Q ( {t, s} )$, $\gamma ( t )$, and $W ( t )$ as in (3.4), (3.15), (3.16), and (3.17), respectively. The same as in the proof of (3.18) in Theorem 3.2, we know that inequality (3.18) remains true here. By (3.18), we know that

3.25 $$ \begin{aligned}[b] &\int _{t_{1}}^{t} K\rho (s) H(t,s)q(s)\,ds\\ &\quad \le H(t,t_{1})\omega (t _{1}) \\ &\qquad {} - \int _{t_{1}}^{t} \bigl\{ \omega ^{2}(s) W(s)H(t,s)+\omega (s) \bigl( {h ( {t, s} ) \sqrt[2]{H(t,s)}-H(t,s)\gamma (s)} \bigr) \bigr\} \,ds. \end{aligned} $$

From inequality (3.25), it follows that

3.26 $$ \begin{aligned}[b] & \limsup_{t\to \infty } \frac{1}{H (t, t_{1} )} \int _{t_{1}}^{t} \biggl[ K H(t,s) \rho (s) q ( s )- \frac{Q ^{2} (t, s )}{4W ( s )} \biggr]\,ds \\ & \quad \le \omega ( t_{1} )-\lim_{t\to +\infty }\inf \frac{1}{H ( {t, t_{1} } )} \int _{t_{1}} ^{t} \biggl[ \sqrt[2]{H(t,s)W(s)}\omega (s)+\frac{Q(t,s)}{2\sqrt[2]{W(s)}} \biggr]^{2}\,ds \\ & \quad = H(t,t_{1})\omega (t_{1})- \int _{t_{1}} ^{t} \bigl\{ \bigl(\omega (s) \sqrt[2]{W(s)H(t,s)} \bigr)^{2} \\ & \qquad {}+\sqrt[2]{H(t,s)}\omega (s) \bigl(h(t,s)-\sqrt[2]{H(t,s)}\gamma (s) \bigr) \bigr\} \,ds \\ & \quad = H ( t, t_{1} ) \omega ( t_{1} )- \int _{t_{1}} ^{t} \biggl\{ \biggl(\omega (s) \sqrt[2]{W(s)H(t,s)}+ \frac{1}{2}\frac{h(t,s)-\sqrt[2]{H(t,s)}\gamma (s)}{\sqrt[2]{W(s)}} \biggr)^{2} \\ &\qquad {}-\frac{ ( {h ( {t, s} )-\sqrt[2]{H ( {t, s} )} \gamma ( s )} )^{2}}{4W(s)} \biggr\} \,ds \\ & \quad = H(t, t_{1})\omega (t_{1})- \int _{t_{1}}^{t} \biggl(\omega (s) \sqrt[2]{W(s)H(t,s)}+\frac{1}{2}\frac{h(t,s)-\sqrt[2]{H(t,s)}\gamma (s)}{ \sqrt[2]{W(s)}} \biggr)^{2} \,ds \\ &\qquad {} + \int _{t_{1}} ^{t} \frac{ ( {h ( {t, s} )-\sqrt[2]{H ( {t, s} )}\gamma ( s )} )^{2}}{4W(s)} \,ds \\ & \quad \le H ( t, t_{1} ) \omega ( t_{1} )- \int _{t_{1}} ^{t} \biggl[\sqrt[2]{H(t,s)W(s)}\omega (s) +\frac{1}{2}\frac{Q(t,s)}{ \sqrt[2]{W(s)}} \biggr]^{2}\,ds+ \int _{t_{1}}^{t}\frac{Q^{2}(t,s)}{4W(s)}\,ds. \end{aligned} $$

Combining (3.24) and (3.26), we deduce that

$$ \omega (t_{1})\ge \varPsi (t_{1})+\lim _{t\to +\infty }\inf \frac{1}{H(t,t _{1})} \int _{t_{1}}^{t} \biggl[\sqrt[2]{H(t,s)W(s)}\omega (s)+\frac{Q(t,s)}{ \sqrt[2]{2W(s)}} \biggr]^{2}\,ds, $$

i.e.,

3.27 $$ \begin{aligned} 0 & \le \liminf_{t\to +\infty } \frac{1}{H(t,t_{1})} \int _{t _{1}} ^{t} \biggl[\sqrt[2]{H(t,s)W(s)}\omega (s)+\frac{Q(t,s)}{ \sqrt[2]{2W(s)}} \biggr]^{2}\,ds \\ & \le \omega (t_{1})-\varPsi (t_{1})< \infty . \end{aligned} $$

We define two functions *u* and *v* as follows:

3.28 $$ u(t)=\frac{1}{H(t,t_{1})} \int _{t_{1}}^{t} H(t,s) W(s)\omega ^{2}(s)\,ds $$

and

3.29 $$ v(t)=\frac{1}{H(t,t_{1})} \int _{t_{1}} ^{t} \sqrt[2]{H(t,s)}Q(t,s) \omega (s)\,ds. $$

Combining (3.22) and (3.27), we deduce that

3.30 $$ \lim_{t\to +\infty } \inf \bigl[ {u ( t )+v ( t )} \bigr]< \infty . $$

Using (3.30), we claim and prove the following inequality:

3.31 $$ \int _{t_{1}}^{\infty } W(s)\omega ^{2}(s)\,ds < \infty . $$

We prove it by using a contradiction argument. If (3.31) were not true, then

3.32 $$ \int _{t_{1}}^{\infty } W(s)\omega ^{2}(s)\,ds = \infty . $$

By (3.21), there exists a positive constant *χ* such that

3.33 $$ \inf_{s\ge T} \biggl[\lim_{t\to +\infty } \inf \frac{H(t,s)}{H(t,T)} \biggr]> \chi . $$

Let *δ* be an arbitrary positive number. By (3.32), we know that there exists $t_{2} \ge t_{1} $ such that, for $t\ge t_{2}$,

3.34 $$ \int _{t_{1}}^{t} W(s)\omega ^{2}(s)\,ds\ge \frac{\delta }{\chi }. $$

By (3.28), we know that, for $t\ge t_{2}$,

3.35 $$ \begin{aligned}[b] u(t) & = \frac{1}{H(t,t_{1})} \int _{t_{1}}^{t} H(t,s)\frac{d}{\,ds} \biggl[ \int _{t_{1}}^{s} W(u)\omega ^{2}(u)\,du \biggr] \,ds \\ & = \frac{1}{H(t,t_{1})} \int _{t_{1}} ^{t}-\frac{\partial H(t,s)}{ \partial s} \biggl[ \int _{t_{1}}^{s} W(u)\omega ^{2}(u)\,du \biggr]\,ds \\ &\ge \frac{1}{H(t,t_{1})} \int _{t_{2}} ^{t}-\frac{\partial H(t,s)}{ \partial s} \biggl[ \int _{t_{1}}^{s} W(u)\omega ^{2}(u)\,du \biggr]\,ds \\ &\ge \frac{\delta }{\chi }\frac{1}{H(t,t_{1})} \int _{t_{2}}^{t} -\frac{ \partial H(t,s)}{\partial s}\,ds \\ & = \frac{\delta }{\chi }\frac{H(t,t_{2})}{H(t,t_{1})}. \end{aligned} $$

By (3.21), we know that there exists $t_{3} \ge t_{2} $ such that, for $t\ge t_{3} $,

3.36 $$ \frac{H(t,t_{2})}{H(t,t_{1})}\ge \chi . $$

Combining (3.35) and (3.36), we know $u ( t ) \ge \delta $ for $t\ge t_{3} $. From the arbitrariness of *δ*, we conclude that

3.37 $$ \lim_{t\to +\infty } u ( t )=\infty , $$

which is a contradiction with (3.30), and we have proved claim (3.31).

From (3.30), we can choose a sequence $\{t_{n}\}_{n=1}^{ \infty } \subset (t_{0} ,\infty )$ satisfying $\lim_{n\to +\infty } t _{n} = +\infty $ such that

3.38 $$ u(t_{n})+v(t_{n})\le M \quad \text{for } n=1,2,3,\ldots , $$

for some positive number *M*.

Combining (3.28) and (3.32), we obtain

3.39 $$ \lim_{n \to +\infty } u ( {t_{n} } )=+\infty . $$

Combining (3.38) and (3.39), we get

3.40 $$ \lim_{n\to +\infty } v(t_{n})=-\infty . $$

By (3.38) and (3.39), we know there exists a positive integer *N* such that, for $n\ge N$,

3.41 $$ 1+\frac{v(t_{n})}{u(t_{n})}\le \frac{M}{u(t_{n})}< \frac{1}{2} \quad \text{or} \quad \frac{v(t_{n})}{u(t_{n})}< -\frac{1}{2}. $$

Combining (3.39), (3.40), and (3.41), we obtain the following:

3.42 $$ \lim_{n\to +\infty } \frac{v^{2}(t_{n})}{u(t_{n})}=+\infty . $$

On the other hand, by using the Schwarz inequality, we obtain

$$ \begin{aligned} v^{2}(t_{n}) & = \biggl[\frac{1}{H(t_{n},t_{1})} \int _{t_{1}}^{t_{n}} \sqrt[2]{H(t _{n},s)} Q(t_{n},s)\omega (s)\,ds \biggr]^{2} \\ &\le \frac{1}{H^{2}(t_{n},t_{1})} \int _{t_{1}}^{t_{n}} H(t_{n},s)W(s) \omega ^{2}(s)\,ds \int _{t_{1}}^{t_{n}} \frac{Q^{2}(t_{n},\varphi )}{W(s)}\,d\varphi \\ & =u(t_{n})\frac{1}{H(t_{n},t_{1})} \int _{t_{1}}^{t_{n}} \frac{Q^{2}(t _{n},s)}{W(s)}\,ds \end{aligned} $$

for any arbitrary integer *n*. By the above inequality, we get

3.43 $$ \frac{v^{2}(t_{n})}{u(t_{n})}\le \frac{1}{H(t_{n},t_{1})} \int _{t_{1}} ^{t_{n}} \frac{Q^{2}(t_{n},s)}{W(s)}\,ds. $$

Combining (3.42) and (3.43), we deduce that

3.44 $$ \lim_{n\to +\infty } \frac{1}{H(t_{n},t_{1})} \int _{t_{1}}^{t_{n}} \frac{Q ^{2}(t_{n},s)}{W(s)}\,ds \to +\infty . $$

By the arbitrariness of the sequence $\{ {t_{n} } \}_{n=1} ^{\infty }$, it is obvious that (3.44) contradicts condition (3.22) in Theorem 3.3.

The case for $y^{ ( n )}(t)<0$ is similar to the proof of Theorem 3.1, and we omit the details here. We thus have completed the proof of Theorem 3.3. □

## Some applications of the asymptotic dichotomy

In this section, we give some examples to illustrate the applications of the asymptotic dichotomy proved in the previous section.

### Example 4.1

Consider the fourth-order delay differential equation

4.1 $$ y^{ ( 4 )} ( t )+\frac{1}{8t^{2}}{y}'' ( t )+ \biggl( {1-\frac{1}{8t^{2}}} \biggr)y ( {t-\pi } )=0 \quad \text{for } t>\pi + 1. $$

Here, comparing with equation (1.1), we see $p(t)=\frac{1}{8t ^{2}}$, $q(t)=1-\frac{1}{8t^{2}}$, $f ( u )=u$ with $K=1$ and $g(t)=t-\pi $.

It is easy to verify that equation (4.1) satisfies condition (3.2) and that the equation ${z}''+\frac{1}{8t^{2}}z=0$ is non-oscillatory. We now take $\rho =1$ and then obtain

$$ \begin{aligned} & \limsup_{t\to + \infty } \int _{T}^{t} K\rho (s)q(s)-\frac{2^{2}2! (\rho '(s)-p(s)\rho (s)(s-g(s)) )^{2}}{4g^{2}(s)g'(s)\rho (s)}\,ds \\ &\quad = \limsup_{t\to + \infty } \int _{1}^{t} \biggl\{ 1-\frac{1}{8s ^{2}}- \frac{2}{(s-\pi )^{2}} \biggl( -\frac{1}{8s^{2}}(\pi ) \biggr)^{2} \biggr\} \,ds =+\infty . \end{aligned} $$

Hence condition (3.3) in Theorem 3.1 holds, and we can use Theorem 3.1 to conclude that all the solutions of equation (4.1) are oscillatory or approaching to zero as $t\to +\infty $. In fact, we can verify that $y ( t )=-\cos t$ is a solution of equation (4.1) which is oscillatory.

### Example 4.2

Consider the fifth-order delay differential equation in the following:

4.2 $$ y^{ ( 5 )} ( t )+e^{-2t+2}{y}''' ( t )+ \frac{1}{e}y ( {t-1} ) \bigl( {1+y^{2} ( {t-1} )} \bigr)=0 \quad \text{for } t > 3, $$

where comparing with equation (1.1), we take $p(t)=e^{-2t+2}$, $q(t)=\frac{1}{e}$, $f ( u )=u ( {1+u^{2}} )$ with $K=1$ and $g(t)=t-1$.

It is easy to verify that equation (4.2) satisfies condition (3.2) and the equation ${z}''+e^{-2t+2}z=0$ is non-oscillatory. We choose $H ( {t,s} )= ( {\frac{ ( {t-2} ) ^{3}}{6}-\frac{ ( {s-2} )^{3}}{6}} )^{2}$ and $\rho =1$ and verify that

$$ \begin{aligned} &\limsup_{t\to + \infty } \frac{1}{H(t,T)} \int _{T}^{t} \biggl(\mathit{KH}(t,s)q(s)- \frac{Q ^{2}(t,s)}{4W(s)H(t,s)} \biggr)\,ds \\ &\quad = \limsup_{t\to + \infty } \frac{1}{ [\frac{(t-2)^{3}}{6} ]^{2}} \int _{2}^{t} \biggl\{ \biggl[\frac{(t-2)^{3}}{6}- \frac{(s-2)^{3}}{6} \biggr]^{2} \biggl(\frac{1}{e} \biggr) \\ &\qquad {}-\frac{ \{{6 ({s-2} )^{2}+ [\frac{(t-2)^{3}}{6}- \frac{(s-2)^{3}}{6} ]e^{-2s+2}} \}^{2}}{ \frac{(s-1)^{3}}{12} [ {\frac{(t-2)^{3}}{6}-\frac{(s-2)^{3}}{6}} ]^{2}} \biggr\} \,ds=+\infty . \end{aligned} $$

Therefore condition (3.14) in Theorem 3.2 holds, and we use Theorem 3.2 to conclude that all the solutions of equation (4.2) are oscillatory or approaching to zero as $t\to +\infty $. It is easy to verify that $y ( t )=e^{-t}$ is a solution of equation (4.2) which is approaching to zero as $t\to +\infty $.
